# Association between Biomarkers of Oxidative Stress and Inflammation with Cardiac Necrosis and Heart Failure in Non-ST Segment Elevation Myocardial Infarction Patients and Various Degrees of Kidney Function

**DOI:** 10.1155/2021/3090120

**Published:** 2021-11-01

**Authors:** Stefanos Roumeliotis, Andrej Veljkovic, Panagiotis I. Georgianos, Gordana Lazarevic, Zoran Perisic, Jovan Hadzi-Djokic, Vassilios Liakopoulos, Gordana Kocic

**Affiliations:** ^1^Division of Nephrology and Hypertension, 1st Department of Internal Medicine, AHEPA Hospital, School of Medicine, Aristotle University of Thessaloniki, 54636 Thessaloniki, Greece; ^2^Department for Biochemistry, Faculty of Medicine, University of Nis, 18000 Nis, Serbia; ^3^Division of Cardiology, Clinical Center in Nis, Faculty of Medicine, University of Nis, 18000 Nis, Serbia; ^4^Serbian Academy of Sciences and Arts, 11000 Belgrade, Serbia

## Abstract

The aim of this study was to explore the possible association between markers of inflammation and oxidative stress (OS) and markers of cardiac function and necrosis in 100 NSTEMI (non-ST-elevation myocardial infarction) patients with various degrees of kidney dysfunction. At admission, ejection fraction (EF), brain natriuretic peptide (BNP), troponin (TnI), creatinine phosphokinase (CPK), alanine transaminase (ALT), aspartate transaminase (AST), high-sensitive C-reactive protein (hs-CRP), interleukins 6 and 10 (IL-6, IL10), myeloperoxidase (MPO), transforming growth factor beta (TGF-*β*1), glomerular filtration rate (GFR), and albuminuria were assessed. Study participants were divided into 2 subgroups based on the median level of EF. Compared to the high, patients in the low EF group had higher GFR, BNP, CPK, hs-CRP, IL-10, IL-6, and MPO values and lower albuminuria levels. The levels of EF decreased in parallel with the progression of CKD, whereas the levels of BNP, IL-6, and TGF-*β* were significantly higher in late stages of CKD. Spearman's rho correlation analysis showed that EF was inversely correlated with MPO (*r* = −0.20, *p* = 0.05) BNP (*r* = −0.30, *p* = 0.002), hs-CRP (*r* = −0.38, *p* < 0.0001), IL-10 (*r* = −0.30, *p* = 0.003), and IL-6 (*r* = −0.24, *p* = 0.02) and positively with GFR (*r* = 0.27, *p* = 0.008). TnI was correlated with CPK (*r* = 0.44, *p* < 0.0001), CPK-MB (*r* = 0.31, *p* = 0.002), ALT (*r* = 0.50, *p* < 0.0001), AST (*r* = 0.29, *p* = 0.004), IL-10 (*r* = 0.22, *p* = 0.03), and MPO (*r* = −0.28, *p* = 0.006). In multivariate regression analysis, only BNP (*β* = −0.011, *p* = 0.004), hs-CRP (*β* = −0.11, *p* = 0.001), and GFR (*β* = 0.12, *p* = 0.0029) were independent determinants of EF. Similarly, MPO (*β* = −1.69, *p* = 0.02), IL-10 (*β* = 0.15, *p* = 0.006), and AST (*β* = 0.04, *p* = 0.001) were the 3 major determinants of TnI. Based on these associations, we built a predictive model including markers of inflammation and OS (MPO, IL-10, and hs-CRP) to identify patients with the most severe cardiac injury (combined EF below median and troponin above median values). Receiver-operator characteristic (ROC) analysis showed that the area under the ROC curve of this model to detect patients with low EF and high TnI was 0.67 (*p* = 0.015, 95%confidence interval = 0.53‐0.81).

## 1. Introduction

Acute coronary syndrome (ACS), one of the most common manifestations of atherosclerosis, is caused by the rupture or erosion of unstable atherosclerotic plaque. Registry data show that ACS is more prevalent in patients with non-ST-segment elevation (NSTE-ACS) than in those with ST-segment elevation myocardial infarction (STEMI) [1, 2]. Although in-hospital mortality is higher in patients with STEMI compared to non-ST-elevation myocardial infarction (NSTEMI) at the time of admission, the 6-month mortality rates are similar in both entities. Most importantly, after 4 years of follow-up, compared to STEMI, NSTEMI patients have a twofold increase in overall mortality [3]. This may be explained by the fact that patients with NSTEMI-ACS are older and suffer from more comorbidities, such as diabetes and chronic kidney disease (CKD), endothelial dysfunction, and inflammation [4].

Low-grade systemic inflammation and oxidative stress (OS) play a key role in the onset and development of atherosclerosis [5–7]. These interrelated entities—which are significantly deranged in CKD—upregulate cytokines and growth factors resulting in endothelial damage and dysfunction, which is the hallmark of atherosclerosis [6]. In NSTEMI-ACS patients, inflammatory cytokines and markers are predictors of cardiovascular (CV) risk and OS amplifies this association. This could be attributed to the fact that these molecules do not reflect inflammation solely, but may significantly contribute to plaque rupture [8]. However, a slightly different inflammatory pattern was documented between STEMI and NSTEMI patients on account of increased inflammatory response in STEMI [9]. OS also plays a central role in the pathogenesis and progression of atherosclerosis. The enzyme myeloperoxidase (MPO) is secreted by activated neutrophils and monocytes in atherosclerotic plaque [10] and is a critical mediator of destabilization and rupture of atherosclerotic plaque, leading to ACS [11, 12]. Recent data show that MPO may represent a useful diagnostic and prognostic tool in patients with endothelial dysfunction and ACS [13], over a period of 30 days up to six months, even in patients with negative cardiac troponin T (TnT) values and in the absence of myocardial necrosis.

There are several markers of cardiac injury and function in patients with NSTEMI-ACS. Neurohumoral activity of the heart can be determined by measuring natriuretic peptides, BNP (brain natriuretic peptide), and N-terminal fragment of its prohormone (N-terminal proBNP, NT-proBNP). These are highly sensitive markers of left ventricular dysfunction, increased end-diastolic pressure, and left ventricular wall stress. In patients with NSTEMI-ACS, these markers are significant predictors of death when assessed at admission [14], but their predictive ability for mortality is significantly increased, when these biomarkers are assessed a few days after symptom onset [15]. BNP, a counterregulatory hormone involved in the regulation of blood pressure and body fluid volume, is elevated in patients with congestive heart failure (HF) proportionally to the degree of left ventricular dysfunction, severity of the cardiac injury, and the level of ejection fraction (EF). TnI—along with CPK (creatine phosphokinase), CPK-MB (creatine phosphokinase-MB), ALT (alanine transaminase), and AST (aspartate transaminase)—reflect myocardial necrosis. The isoforms of CPK and AST differ significantly in their sensitivity, specificity, and dynamics [16]. Both BNP and Tnl have been established as short- and long-term prognostic biomarkers in patients with NSTEMI.

The current multiomic approach of cardiac-specific biomarkers should be able to distinguish the diagnostic tests for structural or functional cardiac dysfunction, the extent of myocardial injury, necrosis, and survival rate. A significant variability in the presence of kidney dysfunction should be considered as well. Despite the pivotal role of inflammation and OS in the pathogenesis and progression of atherosclerosis and CVD, most of inflammation and OS markers have not been utilized in everyday clinical practice. The identification of novel tissue-specific markers with both high sensitivity/specificity for premature detection and strong long-term prognostic significant is a major goal of future research.

The aim of this study was to investigate possible associations of OS and inflammatory biomarkers with cardiac injury and function among patients presenting with NSTEMI-ACS. A particular aim of the present work was to explore whether the severity of kidney dysfunction modifies these associations.

## 2. Materials and Methods

### 2.1. Patients

This prospective cohort study was conducted from 31 January 2018 until 1 February 2019. We enrolled 100 consecutive patients presenting with NSTEMI in the emergency department and admitted for treatment in the Clinic for Cardiovascular Diseases of the University Clinical Center in Nis (Serbia) during the study period. The inclusion criteria for our study were a voluntary informed consent before enrolment, NSTEMI diagnosis (determined by clinical and electrocardiogram examination and confirmed by laboratory analysis), and voluntary, informed consent to the proposed methods of examination and treatment. Patients that refused to be engaged in the study and those with verified alcohol and/or illicit drug abuse were excluded from this study, on the assumption that compliance might not be satisfactory, as described before [17].

The diagnosis of ACS was based on patients' history, clinical, electrocardiographic, and biochemical criteria. A special reference was undertaken to present risk factors for CV disease and present comorbidities (age, hypertension, dyslipidemia, diabetes mellitus, obesity, and cigarette smoking), both personal and family history for CV disease.

Clinical examination of patients, electrocardiogram, echocardiography, and coronary angiography were performed at the Cardiology and Cardiovascular Disease Clinic.

### 2.2. Invasive and Noninvasive Imaging Evaluation

All patients underwent echocardiography on the first day of hospitalization, immediately after admission, and LVEF was assessed noninvasively, using standard methods (Teichholz method to Simpson's method) as described before [18, 19]. We performed invasive coronary angiography in patients who were informed in detail about all aspects of the angiographic examination (importance, procedure, potential risks, and therapeutic possibilities) and provided informed consent accepting this diagnostic method and treatment with one of the revascularization procedures. The attending physician assessed the periprocedural risk based on clinical status, laboratory analyses, and comorbidities. Coronary angiography was performed within 2 hours of admission in patients with a very high ischemic risk. High ischemic risk included refractory angina pectoris, heart failure, severe heart rhythm disorders, or hemodynamic instability. Coronary angiography was performed within 24 hours in patients with a GRACE score higher than 140 or with at least one primary high-risk criterion, and within 72 hours in patients with at least one high-risk criterion and recurrent angina. According to these scores, 51 of the patients underwent percutaneous coronary intervention (PCI). Revascularization strategy (PCI or coronary artery bypass grafting-CABG) was further determined by attending physicians based on clinical status, angiographic characteristics of the lesion determined by SYNTAX score, angiographic findings of disease severity, comorbidities, and patients' compliance with the proposed procedure.

The study was conducted in accordance with the Declaration of Helsinki, and it was approved by the Ethics Committee of Clinical Centre in Niš, as a part of the project number 175092 (Decision No. 17776/8).

### 2.3. Laboratory Analyses

Standard laboratory analyses and assessment of markers of inflammation, OS, cardiac necrosis, and cardiac function were performed at the Center for Medical Biochemistry and at the Laboratory for Immunology of UCC Nis. Standard biochemical analyses were performed on a multichannel biochemical analyzer (OLYMPUS AU400e® Chemistry Immuno Analyzer), by using manufacturer reagents, for the following parameters: glucose (enzymatic UV test with hexokinase, expressed in mmoL/L), HbA1c (glycated hemoglobin) (immunoinhibition test, expressed in %), total cholesterol (TC, expressed in mmoL/L by CHOD-PAP method), HDL (high-density lipoprotein) cholesterol (HDL-C enzymatic color test, expressed in mmoL/L), LDL (low-density lipoprotein) cholesterol (LDL-C determined by Friedwald's formula (unless the triglyceride value was greater than 3.96 mmol, expressed in mmoL/L), urea (GLDH method using a kinetic UV test, expressed in mmoL/L), creatinine (kinetic color test, expressed in *μ*mol/L), and AST (kinetic UV test, expressed in IU/L).

Serum TnI concentration was determined with an AxSYM analyzer (Abbott), using the original AxSYM Troponin I ADV immunoassay “sandwich” technique with monoclonal antihuman anti-cTnI antibodies, expressed in *μ*g/L. Serum myoglobin concentration was determined by immunoturbidimetric assay, by using the original manufacturer (Olympus) myoglobin immunoassay “sandwich” technique with monoclonal antihuman antimyoglobin antibodies, expressed in *μ*g/L.

Serum CPK and CPK-MB were determined on Olympus analyzer using the original Beckman Coulter kinetic UV tests; serum CPK-MB mass concentration was determined with an AxSYM analyzer (Abbott), using the original AxSYM CPK-MB immunoassay “sandwich” technique with monoclonal antihuman anti-CPKMB antibodies, expressed in IU/L and *μ*g/L.

Hs-CRP (high-sensitive C-reactive protein) was determined by immunoturbidimetric method on the Olympus system, using highly sensitive application (0.08-80 mg/L), with latex particles coated with antihuman CRP antibodies. Fibrinogen was determined by immunoturbidimetric method, using saturated Parfrontier solution. Normal values of fibrinogen are considered in the reference range of 2.0-4.0 g/L. Serum IL-6 (interleukin-6) was determined by ELISA Human IL-6 immunoassay (Quantikine HS; R&D Systems Inc, Minneapolis, Minnesota, USA), based on a sandwich technique with polyclonal antihuman antibodies that bind to recombinant human IL-6. The minimum detectable dose for IL-6 is in the range of 0.016–0.110 pg/mL (mean minimum detectable dose 0.09 pg/mL). Serum IL-10 (interleukin-10) was determined by ELISA Human IL-10 immunoassay (Quantikine HS; R&D Systems Inc., Minneapolis, Minnesota, USA), based on a sandwich technique with polyclonal antihuman antibodies that bind to recombinant human IL-10. The minimum detectable dose is <0.5 pg/mL. Serum MPO was determined by MPO ELISA immunoassay (Quantikine HS; R&D Systems Inc., Minneapolis, Minnesota, USA), based on a “sandwich” technique with two polyclonal antihuman antibodies, which bind to MPO. The minimum detectable dose is 0.2-10 ng/mL and sensitivity of 0.062 ng/mL. Serum TGF-*β*1 (transforming growth factor beta) was determined by ELISA Human TGF-*β*1 immunoassay (Quantikine HS; R&D Systems Inc., Minneapolis, Minnesota, USA), based on a “sandwich” technique with polyclonal antihuman antibodies. The minimum detectable dose is <0.1 pg/mL. BNP was evaluated in the plasma by an AxSYM analyzer (Abbott), using an original AxSYM BNP immunoassay based on a “sandwich” technique with monoclonal antihuman BNP antibodies and BNP values are expressed in pmol/L.

GFR (glomerular filtration rate) was calculated using the chronic kidney disease epidemiology collaboration (CKD-EPI) equation [20], and the classification of CKD stages was based on the criteria of the Clinical Practice Guidelines for Chronic Kidney Disease from the National Kidney Foundation–Kidney Disease Outcomes Quality Initiative [21]. The presence of albuminuria (urine albumin to creatinine ratio—ACR) was evaluated in a morning spot urine sample, as described elsewhere [22]. Albuminuria was determined by immunoturbidimetric method, using the original manufacturer (Olympus) reagent.

### 2.4. Statistical Analysis

We tested the data for normality by the Kolmogorov-Smirnov test. Binary data are presented as frequencies (%), normally distributed variables are expressed as mean ± SD, whereas nonnormally distributed are presented as median (interquartile range). For the description of baseline characteristics, all patients were divided into 2 groups, according to the median value of EF (i.e., below and above or equal 53%). Comparisons for nonnormally distributed variables among groups of EF, CKD stages, and albuminuria categories were performed with Mann-Whitney test or Kruskal-Wallis test as appropriate and for categorical variables using *χ*^2^ test. Normally distributed continuous variables were compared by independent *T*-test. Bivariate associations between continuous variables were explored using Spearman's rho correlation coefficient. Correlation analysis of MPO, EF, and troponin with various factors (age, gender, GFR, HbA1c, urea, cholesterol (total, HDL, LDL), *ΒΝ*P, fibrinogen, erythrocytes, leukocytes, *Ε*F, troponin, MPO, CPK, CPK-MB, CRP, ALT, AST, Hb, IL-10, IL-6, TGF-*β*, and albuminuria) was conducted on an exploratory basis to determine possible determinants of MPO, EF, and troponin. Multiple imputations were performed to replace missing data. We performed stepwise multiple regression analysis to identify the most important determinants of EF and troponin (log-transformed), as previously determined by correlation analysis. We divided our patients into 4 categories, according to troponin values (above/below median value) and EF values (above/below median values) as follows: high EF/low troponin, low EF/low troponin, high EF/high troponin, and low EF/high troponin. Based on the regression analyses, to determine patients at the 4^th^ category (with combined low EF and high troponin), we forced IL-10, hs-CRP, and MPO into a predictive model. The discrimination-predictive ability of this model was assessed by the receiver-operating characteristic (ROC) curves. Since impaired kidney function (assessed by GFR and albuminuria) is a significant, but often, clinically underestimated risk factor for atherosclerosis and cardiovascular disease, we divided patients in albuminuria categories and CKD stages based on albuminuria and GFR values, respectively. Albuminuria categories were defined as A1-normally to mildly increased, with ACR ≤ 30 mg/g, A2-moderately increased, with ACR between 30 and 300 mg/g, and A3 as severely increased, with ACR > 300 mg/g. CKD stages were defined as follows: stage 1 with GFR ≥ 90 mL/min, stage 2 with GFR between 60 and 89 mL/min, stage 3 with GFR between 30 and 59 mL/min, stages 4 with GFR between 15 and 29 mL/min, and stage 5 with GFR under 15 mL/min. CKD stages 4 and 5 were combined, due to the small number of patients. Statistically significance was set at a *p* value of <0.05. Statistical analyses were performed by using the IBM Statistical Package for Social Sciences-SPSS 18.0 for Windows, Chicago, Illinois, USA.

## 3. Results

We enrolled 100 consecutive patients presenting with NSTEMI in the emergency department and admitted for treatment. The mean age of our patients was 66.54 ± 11.44 years, 73% had history of preexisting hypertension, 71% dyslipidemia, and 42% received treatment for type 2 diabetes mellitus. Sixty-two percent were overweight or obese and 43% had a history of smoking. Regarding the history of CV disease, half of the patients had a history of angina, 34% had already experienced a previous MI and 14% had previously undergone PCI. Coronary angiography revealed single-vessel CAD in 31 patients, dual-vessel disease in 11 patients, and three-vessel disease in 9 patients, and twelve patients underwent surgical revascularization of the myocardium (CABG). During the hospitalization for NSTEMI (acute phase), hemodynamic instability occurred in 23 patients, and arrhythmic complications, such as ventricular tachycardia or ventricular fibrillation, were detected in 9 patients. Anthropometric, clinical, and biochemical data of patients with NSTEMI according to median values of EF are presented in Table 1. No significant between-group differences were observed in age, Hb, RBC, WBC, cholesterol (total/LDL/HDL), troponin, ALT, AST, CK-MB, fibrinogen, and TGF-*β* levels. However, patients in the low EF group had significantly higher HbA1c and albuminuria levels and lower GFR values. Compared to the high EF group, patients in the low EF group had higher BNP, CPK, hs-CRP, IL-10, IL-6, and MPO values.

Table 2 shows the correlation matrix of MPO, EF, and troponin with several characteristics of the patients under study. MPO was significantly and negatively correlated with both EF (*r* = −0.20, *p* = 0.05) and troponin (*r* = −0.28, *p* = 0.006) and positively with hs-CRP (*R* = 0.24, *p* = 0.02), IL-6 (*r* = 0.20, *p* = 0.05), and fibrinogen (*r* = 0.31, *p* = 0.002). Besides MPO, EF was inversely correlated with BNP (*r* = −0.30, *p* = 0.002), hs-CRP (*r* = −0.38, *p* < 0.0001), IL-10 (*r* = −0.30, *p* = 0.003), and IL-6 (*r* = −0.24, *p* = 0.02) and positively with GFR (*r* = 0.27, *p* = 0.008). As expected, in the overall population, there was a significant correlation between troponin and CK (*r* = 0.44, *p* < 0.0001), CK-MB (*r* = 0.31, *p* = 0.002), ALT (*r* = 0.50, *p* < 0.0001), and AST (*r* = 0.29, *p* = 0.004). However, no association was found between troponin and EF or BNP. Moreover, troponin was also correlated with IL-10 (*r* = 0.22, *p* = 0.03) and MPO, as mentioned above.

**Figure 1:**
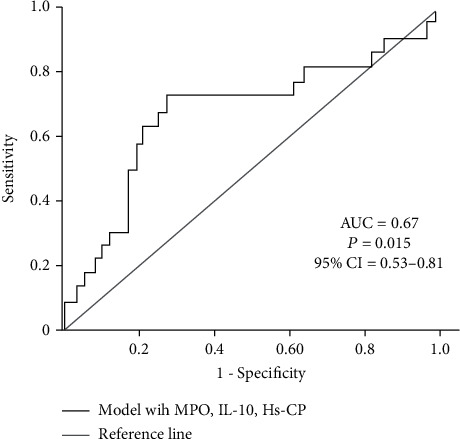
Receiver-operating characteristic curves showing the performance of MPO, IL-10, and Hs-CRP in predicting low EF and high troponin values, in NSTEMI patients.

To determine independent determinants of EF and troponin, we performed multivariate, stepwise linear regression analysis (Table 3) including all the variables exhibiting a significant association with EF (BNP, GFR, hs-CRP, IL-6, IL-10, and MPO) and troponin (MPO, IL-10, AST, ALT, CK, and CK-MB) in the univariate models, respectively. In this analysis, BNP (*β* = −0.011, *p* = 0.004), hs-CRP (*β* = −0.11, *p* = 0.001), and GFR (*β* = 0.12, *p* = 0.0029) were the 3 independent determinants of EF. MPO (*β* = −1.69, *p* = 0.02), IL-10 (*β* = 0.15, *p* = 0.006), and AST (*β* = 0.04, *p* = 0.001) were the 3 parameters that continued to be significantly associated with troponin values. CK, CK-MB, and ALT were excluded from the troponin model. Based on these associations, we build a predictive model including markers of inflammation and OS (MPO, IL-10, and hs-CRP) to predict patients with the most severe cardiac injury (4^th^ category of combined EF below median and troponin above median values). ROC curves (Figure 1) showed that the area under the curve (AUC) of this model to predict low EF and high troponin was 0.67 (*p* = 0.015, 95%confidence interval = 0.53‐0.81).

### 3.1. Evaluation of LVEF, OS, and Inflammation according to the Degree of Renal Impairment

Table 4 shows inflammatory and OS biomarkers according to the degree of renal impairment as expressed by albuminuria categories. Of all variables studied (besides GFR, urea, and creatinine), only IL-10 (*p* = 0.02), TGF-*β*1 (*p* = 0.03), and BNP (*p* = 0.03) were significantly different among albuminuria stages; MPO, IL-6, and hs-CRP did not significantly differ among albuminuria categories. The levels of IL-10, TGF-*β*1, and BNP were increased in parallel with the severity of albuminuria.

**Figure 2:**
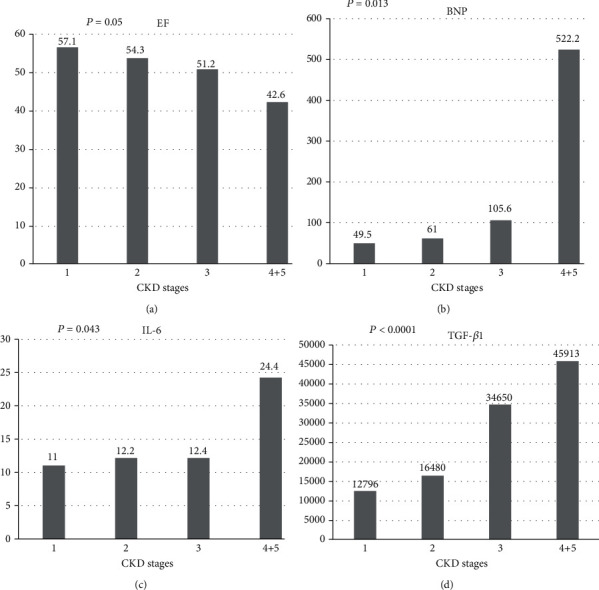
(a) EF at different CKD stages (*p* = 0.05). (b) BNP at different CKD stages (*p* = 0.013). (c) IL-6 at different CKD stages (*p* = 0.043). (d) TGF-*β*1 at different CKD stages (*p* < 0.0001).

We also investigated whether cardiac function/MI and inflammation/OS markers were different among CKD stages. The levels of EF (*p* = 0.05), BNP (*p* = 0.013), IL-6 (*p* = 0.04), and TGF-*β* (*p* < 0.0001) were significantly different among CKD stages.  Figure 2(a) shows that the levels of EF were progressively lower in patients with more severe CKD (EF value was 57.1/54.3/51.2/42.6 in CKD stages 1, 2, 3, 4+5, respectively).  Figure 2(b) shows that the levels of BNP increased in parallel with the progression of CKD (BNP values were 49.5/61/105.6 in stages 1, 2, and 3, respectively, whereas BNP was increased to 522.2 in stage 4+5). Moreover, as compared to patients with stage 1 CKD, the levels of IL-6 were significantly higher in patients with stage 2 (12.2) and 3 (12.4) CKD, whereas the levels of IL-6 were further increased in the group of CKD stage 4+5 (24.4) (as shown in Figure 2(c)). A similar trend was found for TGF-*β*1 across CKD stages: as disease progressed, TGF-*β*1 was significantly increased from 12,796 (stage 1) to 1,6480 (stage 2), 34,650 (stage 3), and to 45,913 (stage 4+5).

## 4. Discussion

Compared to STEMI, patients with NSTEMI-ACS have a twofold increase in 4-year mortality [3]. This might be attributable to the fact that NSTEMI patients typically have a huge burden of comorbidities, such as kidney dysfunction, OS, and inflammation. There are currently several sensitive and reliable cardiac biomarkers (including EF, BNP, NT-proBNP, TnI, ST2, CK, CK-MB, AST, and LDH) that enable the premature detection of cardiac dysfunction, myocardial injury, necrosis, and tissue extracellular matrix remodelling; these biomarkers are often used as surrogate endpoints in randomized trials that explore the efficacy of drug treatment [23].

In patients with ACS, these biomarkers present substantial variability, a phenomenon that is further amplified in those with impaired kidney function. Moreover, despite the fact that the role of biomarkers of inflammation and OS in the development of HF, atherosclerosis, and CAD is undisputed, most of these markers are not routinely assessed in everyday clinical practice. In this study, we aimed to investigate the possible association between markers of inflammation and OS with cardiac markers in a cohort of NSTEMI-ACS patients with different degrees of kidney function.

In our study, we found that EF was progressively reduced and BNP increased along with the severity of CKD. Moreover, IL-6 and TGF-*β* levels were significantly higher in patients with more severe CKD. Patients at the low EF group had significantly lower GFR and higher ACR levels, than those stratified to the high EF group. After adjustment for several confounding factors, multiple regression analysis showed that GFR was a strong and independent determinant of EF. Our findings are in agreement with large epidemiological studies showing that the presence of a mild impairment in kidney function magnifies the risk for cardiovascular complications after an acute MI [24, 25]. The Valsartan in Acute Myocardial Infarction (VALIANT) study enrolled 14,527 patients with complicated acute MI and found that below the eGFR threshold of 81 mL/min, every 10 mL/min decrease in eGFR was associated with 10% higher risk for nonfatal cardiovascular outcomes, irrespectively of treatment [25]. Moreover, severe cardiac failure and low EF were independent predictors of AKI in patients with acute coronary syndrome after PCI [26]. Other studies showed that among 1613 consecutive patients hospitalized for atrial fibrillation, impaired kidney function (defined as eGFR < 60 mL/min) was an independent predictor of EF < 40% (odds ratio 1.94, 95% CI 1.46-2.48), [27]. Similarly, in a large cohort of 12,830 patients with ACS, a GFR below 60 mL/min was an independent predictor of 1-year mortality and prolonged hospitalization [28]. However, although kidney dysfunction is very common in patients with established CV disease, even at 20% of cases, it remains undetected [28]. Moreover, it has been repeatedly shown that despite their high-risk characteristics, patients with acute MI and kidney dysfunction are less frequently subjected to invasive revascularization treatment [28, 29]. The association between impaired kidney function and CV complications in patients with acute MI might be attributed to the upregulation of several factors in the presence of CKD, including the aggravation of OS, inflammation, endothelial dysfunction, vascular calcification, and other growth factors (such as fibrinogen and TGF-*β*) [6, 30]. *Ο*S and inflammation are 2 interrelated pathophysiological processes that are disturbed even at the early stage of CKD, progress along with the decline in GFR, and are further exacerbated by dialysis modalities in those with ESKD [7, 31–34]. As CKD progresses, OS and inflammation trigger endothelial dysfunction and upregulate the expression of various growth factors and cytokines, such as fibrinogen and TGF-*β* [6, 31], which subsequently lead to coronary calcification and atherosclerosis. This is line with our observation that circulating IL-6 and TGF-*β* levels were progressively increased as CKD stages increased. Another indicator of kidney damage is the presence of albuminuria, which has been repeatedly associated with all-cause mortality, hospitalizations, and cardiovascular morbidity and mortality, especially in diabetics, but also in the general population or patients with CV disease [35, 36]. In our study, we found that BNP, IL-10, and TGF-*β* increased significantly across albuminuria categories. Our results are in agreement with other cohort studies reporting a tight, positive correlation between BNP and albuminuria, regardless of GFR in diabetics, [37, 38]. In another study enrolling 1000 primary care patients, BNP was independently associated with GFR, regardless of structural kidney injury [39]. The strong and independent association between BNP and markers of kidney function (GFR and albuminuria) has been also documented in nondiabetic populations, as indicated in the Prevention of Renal and Vascular End-Stage Disease (PREVENT) study [40].

The main finding of our study was that low EF after NSTEMI-ACS was associated with inflammatory markers, such as hs-CRP, IL-10, IL-6, and MPO. MPO and IL-10 were independent predictors of TnI values, whereas hs-CRP predicted EF values. A predictive model consisting only of markers of inflammation and OS (MPO, IL-10, and hs-CRP), and not other cardiac enzymes, provided a satisfactory combination of sensitivity and specificity in the detection of patients with low EF and high Tnl values. During the past twenty years, histologic findings of inflammation in atherosclerotic lesions supported that chronic inflammation plays a central role in the pathogenesis of atherosclerosis and CAD, mainly because it increases the vulnerability of an atherosclerotic plaque to rupture or erosion. The most exhaustingly studied marker of inflammation in CVD and ACS is CRP, an acute phase protein generated in the liver in response to the elevation of cytokines, such as IL-6 and TNF-a. However, cardiomyocyte necrosis might also stimulate CRP formation. Hs-CRP is consistently related to incident CAD and ACS, regardless lipid profile parameters [41], whereas the PEACE trial showed that hs-CRP levels were significantly higher in patients with NSTEMI-ACS than in those with stable angina [42]. In patients with stable CAD, increased hs-CRP even over just 1 mg/L predicted CV events (including MI), and new HF, regardless patients' characteristics and treatment [42]. This trial also showed that hs-CRP could not identify patients with preserved HF and stable CAD. Moreover, data from several trials in STEMI-ACS patients—GUSTO IV ACS [43], TIMI 11A [44], and FRISC (20)—showed that hs-CRP and Tnl were independent and complementary predictors of adverse clinical outcomes, including short- and long-term mortality, whereas the PROVE IT-TIMI 22 trial found that in a cohort of 4162 patients stabilized after ACS, patients with high hs-CRP values had a two-fold increase in the risk of new-onset or worsening HF during 2 years of follow-up [45]. In this study, HF was increased in parallel with increasing hs-CRP and increased hs-CRP was an independent predictor of HF in multivariate analysis. These results are in agreement with our findings that hs-CRP was an independent determinant of low EF. Whether CRP is a marker lacking specificity that is elevated in the inflammatory response or a molecule with direct causal effect in the pathogenesis of atherosclerosis and CAD remains controversial. Early experimental and clinical observations suggested a direct causal association between CRP and CAD, based on the identification of CRP in atherosclerotic lesions and its binding to LDL cholesterol [46], whereas newer, large studies with Mendelian randomization design suggest that the role of CRP may not be causal [47, 48]. Therefore, the determination of the predictive value of CRP and its specificity as a marker for HF, cardiac necrosis, and CV events is of utmost importance. *Α* recent meta-analysis from the Emerging Risk Factors Collaboration included 52 studies and 246,669 persons without history of CVD and found that the addition of CRP to traditional CV risk factors significantly improved the risk stratification by 1.5% [49]. Moreover, the JUPITER trial suggested that statin therapy and lowering of CRP alone might be cardioprotective [50]. However, the trial ended early due to unequivocal evidence in favour of the active-treatment group and did not include a group with reduced hs-CRP; thus, these results should be interpreted with caution. Hs-CRP is an important marker of inflammation, but it does not reflect necrosis (21). The evaluation of hs-CRP might assist in risk stratification in selected populations who are considered to be at modest or high risk for CVD. In our study, we found that hs-CRP predicted HF and combination of low EF and high Tnl with modest specificity and sensitivity.

IL-6 is the only inflammatory cytokine that can trigger the generation of all the acute phase molecules that participate in the inflammatory process, such as CRP, fibrinogen, haptoglobin, serum amyloid A, and 1-chymotrypsin, and therefore, it is believed that plays a central role in the inflammatory response [51]. Circulating IL-6 is an independent predictor of CAD [52]. Compared to patients with stable angina, those presenting with unstable angina have significantly increased levels of this cytokine [53]. Moreover, in patients admitted to the hospital for unstable angina, increased IL-6 levels are predictive of prolonged hospitalization and worse clinical outcomes [54]. Although inflammation has been tightly associated with atherosclerosis, establishing causality for any inflammation marker is difficult. In this direction, two large meta-analyses have shown the pivotal role of IL-6 in the onset and development of inflammation and associated risk for atherosclerosis and CAD [55, 56]. The first meta-analysis included data from 200,000 persons and found that the presence of Asp358Ala allele of the IL6R (IL-6receptor) gene was associated with increased circulating IL-6R, reduced CRP levels, and a 3.4% reduced risk for developing CAD [55]. The other study followed a Mendelian randomization analysis of 133,000 persons showing that the rs7529229 single-nucleotide polymorphism was associated with higher IL-6R and reduced CRP levels and a 5% reduced risk for CAD [56]. Both studies support the notion that IL-6 and CRP have a direct, causal association with CAD, and therefore, these molecules might be novel, future therapeutic targets to prevent CAD.

IL-10, a cytokine with anti-inflammatory properties, has been suggested to protect from atherosclerotic lesion formation and rupture [57]. Similar results to these experimental data were reported by clinical trials showing that patients with ACS have significantly reduced IL-10 levels than those with stable angina [58]. Moreover, in ACS populations, reduced IL-10 levels were predictive of future CV events, whereas elevated circulating IL-10 was associated with lower risk for CV disease [59, 60]. A meta-analysis of 12 studies (involving a total of 5882 patients with ACS) also demonstrated a significant association between elevated IL-10 levels and adverse CV events [61]. In our study, we found that in 100 patients admitted in the hospital with NSTEMI-ACS and various degrees of kidney function, a model with MPO, hs-CRP, and IL-10 could detect patients with low EF and high Tnl, and the diagnostic accuracy of this model was 67%. Our results are in agreement with the CAPTURE trial, a prospective study including 1090 patients with NSETMI-ACS. In this study, a multimarker model including IL-10, MPO, Tnl, and placental growth factor successfully predicted mortality and MI over 4 years of follow-up [62]. However, in this study, the model was not tested for its discriminatory predictive power. The authors concluded that the combination of selected markers might add incremental predictive value to risk assessment and stratification in the NSTEMI-ACS population.

MPO is a leukocyte enzyme that is generated in the inflammatory response and triggers oxidation of LDL cholesterol and formation of foam cells. On this basis, MPO is suggested to be a critical mediator in the pathophysiology of formation, growth, destabilization, and rupture of atherosclerotic plaque, leading to ACS [11, 12]. In agreement with these prior observations, we found that MPO was correlated with inflammatory cytokines that are established risk factors of CAD, such as hs-CRP, IL-6, and fibrinogen. Elevated MPO levels are associated with incident CAD and might predict ACS in patients presenting with acute chest pain [63, 64]. Similarly, the MONICA/KORA Augsburg study enrolled 333 CAD patients and 1727 controls who were followed for 11 years. This study confirmed that increased MPO levels were associated with a 70% higher risk for developing CAD, even after adjustment for several traditional risk factors for CVD [65]. Moreover, as CAD progresses from stable disease to NSTEMIA-ACS and MI, MPO is gradually elevated [12]. Coronary stenting might also cause an acute and transient elevation in MPO levels [11] and in ACS patients, MPO might also predict recurrent ischemic events [66].

Besides predicting incident CAD, increased MPO levels have been also associated with severe HF and have been suggested as predictors adverse clinical outcomes in patients with systolic HF [67] Our results are in accordance with these prior finding, since MPO in the present study was shown to be associated with EF and TnI levels. Moreover, MPO was not only an independent predictor of TnI, but along with IL-10 and hs-CRP could precisely detect patients at the quartile of severe cardiac function (assessed by low EF and high TnI).

Our study investigated the association between biomarkers of inflammation and OS and markers of cardiac function and necrosis in patients with NSTEMI-ACS and various degrees of kidney function. Moreover, we proposed a simple model with three markers of inflammation and OS that predicts patients with low EF and high Tnl. However, our study has certain limitations that should be addressed. GFR was assessed on a single measurement of serum creatinine at admission. However, patients with acute NSTEMI often present with AKI, and therefore, the GFR on might not be representative of their actual kidney function at baseline. The observational, cross-sectional design of this study precludes establishment of any causality and the sample size might be perceived as not large enough. Finally, we assessed BNP, albuminuria, and GFR only once at baseline. However, we recognize that these markers often change rapidly over the course of a hospitalization for ACS.

## 5. Conclusions

In conclusion, our study suggests that in patients with NSTEMI-ACS, combination of inflammation and OS markers might have significant predictive ability for detecting cardiac function and necrosis. Larger studies are warranted to fully elucidate the association of these markers with “hard” clinical outcomes in the future.

## Figures and Tables

**Table 1:** Anthropometric and biochemical characteristics of NSTEMI patients at groups above or below the median value of EF (53%). Results for continuous variables are presented as mean (S.D.) or median (range).

*N* = 100	EF < 53% (*N* = 43)	EF ≥ 53% (*N* = 57)	*p*
Age (years)	67.8 (9.8)	65.8 (12.5)	0.46
Gender (M/F)	26/17	23/34	**0.04**
HbA1c (%)	6.1 (1.0)	5.9 (1.12)	**0.02**

Markers of kidney function			
Urea moL/L	8.1 (4.6)	7.0 (3.3)	0.35
GFR (mL/min)	58.6 (21.6)	69.6 (19.5)	**0.03**
Albuminuria (mg/g)	7.0 (0.03-146.7)	2.2 (0.2-55.8)	**0.045**

Total blood count parameters			
Hb (g/L)	131.9 (21.6)	133.9 (18.4)	0.85
RBC (×10^6^/mm^3^)	4.4 (0.7)	4.6 (0.6)	0.06
WBC (×10^3^/mm^3^)	9.3 (2.3)	9.7 (3.5)	0.77

Lipid metabolism parameters			
Total cholesterol (mmoL/L)	5.7 (1.5)	5.7 (1.3)	0.68
LDL cholesterol (mmoL/L)	3.8 (1.4)	3.6 (1.1)	0.84
HDL cholesterol (mmoL/L)	1.2 (0.3)	1.2 (0.3)	0.86

Markers of cardiac function-myocardial infraction			
*Τ*roponin (*μ*g/L)	2.1 (0.12-22.8)	1.5 (0.04-22.8)	0.48
BNP (pmol/L)	150 (8.2-2029)	59.1 (3.6-656.1)	**0.006**
EF (%)	45 (25-52)	60 (53-78)	**<0.0001**
ALT (IU/L)	24 (6-180)	21 (7-88)	0.15
AST (IU/L)	32.1 (5-187)	22 (9-191)	0.11
CPK (IU/L)	139 (11-3188)	101 (3-2266)	**0.08**
CK-MB (IU/L)	28.6 (3.9-390)	24.9 (2.5-438.6)	0.89

Markers of inflammation, OS, and growth factors			
Hs-CRP (mg/dL)	23.1 (1.8-177)	6.9 (0.5-160.2)	**0.003**
IL-10 (pg/mL)	11.3 (12.8)	6.9 (7.6)	**0.05**
IL-6 (pg/mL)	16.6 (15.8)	10.5 (10.8)	**0.05**
MPO (ng/mL)	1.3 (0.01-4.0)	0.9 (0.09-3.4)	**0.04**
Fibrinogen (g/L)	4.8 (0.2-9.0)	4.9 (0.13-13.0)	0.90
TGF-*β*1 (pg/mL)	26226 (3061-103715)	23700 (2104-69964)	0.38

*p* values of Kruskal-Wallis, independent *T*-test or *χ*^2^ test or for differences of variables among EF groups. ALT: alanine transaminase; AST: aspartate transaminase; BNP: brain natriuretic peptide; CPK: creatine phosphokinase; CPK-MB: creatine phosphokinase-MB; EF: ejection fraction; GFR: glomerular filtration rate; Hb: hemoglobin; HbA1c: glycated hemoglobin; HDL: high density lipoprotein; Hs-CRP: high-sensitive C-reactive protein; IL-6: interleukin 6; IL-10: interleukin 10; LDL: low-density lipoprotein; MPO: myeloperoxidase; RBC: red blood cells; TGF-*β*1: transforming growth factor beta; WBC: white blood cells.

**Table 2:** Correlation matrix between MPO and several characteristics of patients with NSTEMI. Values represent Spearman's correlation coefficients.

	MPO		EF		Troponin	
	*r*	*p*	*r*	*p*	*r*	*p*
Age	-0.05	0.60	-0.10	0.33	-0.10	0.32
GFR	-0.08	0.40	0.27	**0.008**	0.08	0.43
Albuminuria	0.03	0.77	-0.13	0.20	-0.12	0.25
EF	-0.20	**0.05**	—	—	-0.11	0.29
BNP	0.05	0.64	-0.30	**0.002**	-0.001	0.99
Troponin	-0.28	**0.006**	-0.11	0.29	—	—
CPK	-0.10	0.33	-0.18	0.08	0.44	**<0.0001**
CPK-MB	-0.19	0.06	-0.10	0.34	0.31	**0.002**
AST	-0.14	0.18	-0.16	0.11	0.50	**<0.0001**
ALT	-0.01	0.92	-0.13	0.20	0.29	**0.004**
HbA1c	0.10	0.37	-0.19	-0.08	-0.16	0.14
Hemoglobin	-0.05	0.62	0.04	0.74	0.08	0.47
RBC	-0.06	0.56	0.18	0.08	-0.08	0.44
WBC	-0.06	0.55	0.07	0.52	0.05	0.61
Total cholesterol	0.06	0.57	-0.06	0.55	-0.07	0.47
LDL cholesterol	-0.07	0.55	-0.13	0.28	0.03	0.83
HDL cholesterol	0.007	0.95	-0.10	0.41	0.05	0.65
Hs-CRP	0.24	**0.02**	-0.38	**<0.0001**	0.02	0.87
TGF*-β1*	0.11	0.29	-0.08	0.43	-0.09	0.36
IL-10	0.12	0.24	-0.30	**0.003**	0.22	**0.03**
IL-6	0.20	**0.05**	-0.24	**0.02**	0.04	0.69
Fibrinogen	0.31	**0.002**	0.06	0.53	-0.18	0.07
MPO	—	**—**	-0.20	**0.05**	-0.28	**0.006**

Correlation is significant at the 0.05 level. ALT: alanine transaminase; AST: aspartate transaminase; BNP: brain natriuretic peptide; CPK: creatine phosphokinase; CPK-MB: creatine phosphokinase-MB; EF: ejection fraction; GFR: glomerular filtration rate; HbA1c: glycated hemoglobin; HDL: high-density lipoprotein; Hs-CRP: high-sensitive C-reactive protein; IL-6: interleukin 6; IL-10: interleukin 10; LDL: low-density lipoprotein; MPO: myeloperoxidase; RBC: red blood cells; TGF-*β*1: transforming growth factor beta; WBC: white blood cells.

**Table 3:** Regression coefficients (*β*) of multiple regression models with dependent variables EF or troponin and independent variables selected based on respective associations.

	*β*	SE	*p*	95% CI
Model for troponin (log transformed)^a^				
MPO	-1.69	0.7	**0.02**	-3.1 to -0.24
IL-10	0.15	0.05	**0.006**	0.04-0.25
AST	0.04	0.01	**0.001**	0.02-0.06
ALT	—	—	0.18	—
CPK	—	—	0.17	—
CPK-MB	—	—	0.56	—
Model for ejection fraction^b^				
BNP	-0.011	0.004	**0.004**	-0.018 to -0.003
Hs-CRP	-0.11	0.03	**0.001**	-0.18 to -0.05
GFR	0.12	0.05	**0.029**	0.012-0.22
IL-10	—	—	0.88	—
IL-6	—	—	0.76	—
MPO	—	—	0.56	—

^a^CPK, CPK-MB, and ALT are excluded from the model. ^b^IL-6, IL-10, and MPO are excluded from the model.

**Table 4:** Inflammatory and OS biomarkers according to the degree of renal impairment as expressed by the level of albuminuria. Results for continuous variables are presented as mean (S.D.) or median (range).

Albuminuria categories				
	A1	A2	A3	*p*
MPO	0.86 (0.09-3.16)	1.16 (0.01-2.72)	1.10 (0.30-4.0)	0.35
Hs-CRP	13.3 (1.6-135.9)	11.9 (0.9-77.4)	18.5 (0.50-177.0)	0.66
IL-6	11.3 (11.1)	11.6 (12.4)	24.1 (18.8)	0.07
IL-10	6.2 (5.5)	9.0 (8.5)	16.5 (20.4)	**0.02**
TGF-*β*1	17937 (3294-69964)	30000 (2104-68200)	34590 (3061-103715)	**0.03**
*ΒΝ*P	60.2 (3.6-561)	94.2 (8.2-941.9)	309.4 (8.7-2029.5)	**0.03**

*p* values of Kruskal-Wallis or independent *T*-test for differences of variables among albuminuria categories. BNP: brain natriuretic peptide; hs-CRP: high-sensitive C-reactive protein; IL-6: interleukin 6; IL-10: interleukin 10; MPO: myeloperoxidase; TGF-*β*1: transforming growth factor beta.

## Data Availability

The data used to support the findings of this study are included within the article.
